# Oligodeoxynucleotides containing unmethylated cytosine-guanine motifs are effective immunostimulants against pneumococcal meningitis in the immunocompetent and neutropenic host

**DOI:** 10.1186/s12974-021-02077-3

**Published:** 2021-02-02

**Authors:** S. Ribes, L. Zacke, S. Nessler, N. Saiepour, E. Avendaño-Guzmán, M. Ballüer, U. K. Hanisch, R. Nau

**Affiliations:** 1 Institute of Neuropathology, University Medical Center, Georg August University Göttingen, Robert-Koch-Straße 40, D-37075 Göttingen, Germany; 2 Department of Geriatrics, Protestant Hospital Göttingen-Weende, Göttingen, Germany

**Keywords:** Oligodeoxynucleotides containing unmethylated cytosine-guanine motifs (CpG ODN), *Streptococcus pneumoniae*, Meningitis, Toll-like receptor (TLR) 9, Interleukin (IL)-12/IL-23p40, Microglia, Macrophage inflammatory protein (MIP)-1α

## Abstract

**Background:**

Bacterial meningitis is a fatal disease with a mortality up to 30% and neurological sequelae in one fourth of survivors. Available vaccines do not fully protect against this lethal disease. Here, we report the protective effect of synthetic oligodeoxynucleotides containing unmethylated cytosine-guanine motifs (CpG ODN) against the most frequent form of bacterial meningitis caused by *Streptococcus pneumoniae*.

**Methods:**

Three days prior to the induction of meningitis by intracerebral injection of *S. pneumoniae* D39, wild-type and Toll-like receptor (TLR9)^−/−^ mice received an intraperitoneal injection of 100 μg CpG ODN or vehicle. To render mice neutropenic, anti-Ly-6G monoclonal antibody was daily administrated starting 4 days before infection with a total of 7 injections. Kaplan-Meier survival analyses and bacteriological studies, in which mice were sacrificed 24 h and 36 h after infection, were performed.

**Results:**

Pre-treatment with 100 μg CpG ODN prolonged survival of immunocompetent and neutropenic wild-type mice but not of TLR9^−/−^ mice. There was a trend towards lower mortality in CpG ODN-treated immunocompetent and neutropenic wild-type mice. CpG ODN caused an increase of IL-12/IL-23p40 levels in the spleen and serum in uninfected animals. The effects of CpG ODN on bacterial concentrations and development of clinical symptoms were associated with an increased number of microglia in the CNS during the early phase of infection. Elevated concentrations of IL-12/IL-23p40 and MIP-1α correlated with lower bacterial concentrations in the blood and spleen during infection.

**Conclusions:**

Pre-conditioning with CpG ODN strengthened the resistance of neutropenic and immunocompetent mice against *S. pneumoniae* meningitis in the presence of TLR9. Administration of CpG ODN decreased bacterial burden in the cerebellum and reduced the degree of bacteremia. Systemic administration of CpG ODN may help to prevent or slow the progression to sepsis of bacterial CNS infections in healthy and immunocompromised individuals even after direct inoculation of bacteria into the intracranial compartments, which can occur after sinusitis, mastoiditis, open head trauma, and surgery, including placement of an external ventricular drain.

## Background

Immunocompromised individuals, patients with underlying diseases, and adults over 65 years are at a high risk of infection caused by *Streptococcus pneumoniae*, in particular pneumonia, sepsis, and meningitis [1]. While pneumococcal vaccines have reduced the incidence of invasive pneumococcal disease (IPD) caused by vaccine serotypes, a substantial increase in carriage and IPD cases due to non-vaccine serotypes have been reported [2–4]. Pneumococcal meningitis is still a life-threatening disease with mortality rates ranging from 24 to 34% in developed countries and up to 51% in low-income regions [5]. In addition, 25% of the survivors present long-term disabling sequelae [5–8]. Neuronal damage is caused by direct bacterial toxicity and the local and systemic inflammatory responses induced upon bacterial recognition [9].

Many bacteria including pneumococci invade the cerebrospinal fluid (CSF) either by spread from adjacent infections or hematogeneously by using different vascular receptors [10]. Microglia are the resident macrophages of the cerebral tissue and in the absence of leukocytes provide a first line of defense against invading bacteria [11]. Microglial cells have a large repertoire of pattern recognition receptors enabling them to sense and eliminate bacteria invading the central nervous system (CNS) parenchyma [11]. There is a bi-directional cross-talk between microglia, cerebral endothelium, and circulating immune cells: microglia can be activated by systemic and local stimuli including cyto- and chemokines and bacterial products released in the CNS or (in regions with a leaky blood–brain barrier) circulating in the blood. Activated microglia release pro-inflammatory cytokines and chemokines involved in the recruitment of white blood cells infiltrating the infected CNS [12–14]. The innate immune system provides the receptors for the induction of an early inflammatory response essential to fight pathogens. Boosting the host’s innate immune response could provide serotype-independent protection against *S. pneumoniae* providing benefit especially to patients with an impaired immune response. Toll-like receptor (TLR) 9 is a key component of the innate defense against pneumococcal infection [15]. Synthetic oligodeoxynucleotides (ODNs) containing unmethylated CpG motifs (CpG) mimic bacterial DNA and are recognized by TLR9 expressed on immune cells [16]. Among three different TLR ligands, CpG ODN was the most effective TLR agonist to stimulate phagocytosis and intracellular killing of non-encapsulated and encapsulated pneumococci by murine microglial cells in vitro [17].

In the present study, we demonstrate the potential of CpG ODN as enhancer of anti-pneumococcal immune responses in both immunocompetent and neutropenic animals, but not in TLR9-deficient mice. CpG ODN priming prolonged the course of pneumococcal meningitis, and CpG ODN-treated mice showed a trend towards lower mortality. Accordingly, CpG ODN-primed animals showed a shaped cytokine response and an improved control of bacterial growth in the brain, blood, and spleen.

## Methods

### Mice

The animal experiments were approved by the Animal Care Committee of the University Hospital of Göttingen and by the *Niedersächsische Landesamt für Verbraucherschutz und Lebensmittelsicherheit* (*LAVES*), Oldenburg, Lower Saxony, Germany (approval number 33.9-42502-04-059/09). C57Bl/6 wild-type (male and female, 2–3 months old) and TLR9-deficient mice (male and female, 2–5 months old) were used in all experiments. The total number of animals used in this project was 190: 177 wild-type mice (176 males, 1 female, 8–14 weeks, body weight at study entry 24.3 ± 3.0 g, purchased from Charles River Laboratory), and 13 TLR9^−/−^ mice on a C57Bl/6 background [16, 17] (11 males, 2 females, 11–22 weeks, body weight at study entry 29.9 ± 4.3 g, bred at the Central Animal Care Facility, University Hospital of Göttingen). The TLR9-deficient mice originally created by S. Akira, Osaka University, were backcrossed on the C57BL/6 J background for at least 10 generations and then bred at the Central Animal Care Facility of the University Medicine Göttingen [16, 18]. Animals were housed in groups up to 8 mice at 22 °C and a humidity of 55% and a 12 h:12 h light:dark cycle and fed a conventional fiber diet (SSNIFF Mouse Breeding Diet, 10-mm pellets, SSNIFF Spezialdiäten GmbH, Soest, Germany). Anesthesia/analgesia was performed with ketamine/xylazine 100/10 mg/kg body weight.

### CpG ODN

The CpG ODN 1668 (CpG class B, 5′ TCC ATG ACG TTC CTG ATG CT, molecular mass 6382.6 g/mol, TIB Molbiol, Berlin, Germany) [17] was used in the present study. CpG ODN was dissolved in distilled water and stored at − 80 °C. CpG ODN was used at the dose of 100 μg per mouse previously effective in mouse infection and tumor models [19–21], dissolved in 34 μl of distilled water and diluted in 200 μl phosphate-buffered saline (PBS). As in the previous study demonstrating its efficacy in experimental *E. coli* meningitis [21], CpG was administered as a single dose intraperitoneally (ip) 3 days prior to infection. The buffer group received the same amount (34 μl) of distilled water in 200 μl PBS. The injection of CpG oligonucleotides caused a temporary weight loss (48 h) in immunocompetent and neutropenic wild-type mice [weight loss 24 h after injection, 2.9 (1.9/4.0) and 5.6 (4.6/6.3)%, respectively; data presented as medians (25th/75th percentiles)], whereas mice receiving the same volume of PBS slightly gained weight (CpG- versus PBS-treated mice of both experiments: *P* < 0.0001, Mann-Whitney *U* test).

### Bacteria

The *S. pneumoniae* strain D39 (encapsulated, serotype 2), a kind gift of Prof. Sven Hammerschmidt (University of Greifswald, Germany), was used in all experiments. Bacteria were grown overnight on blood agar plates, harvested in 0.9% saline and stored at − 80 °C. Frozen aliquots were used for the experiments and diluted with 0.9% saline to the required bacterial concentration.

### Time course of the experiments

**Fig. 1:**
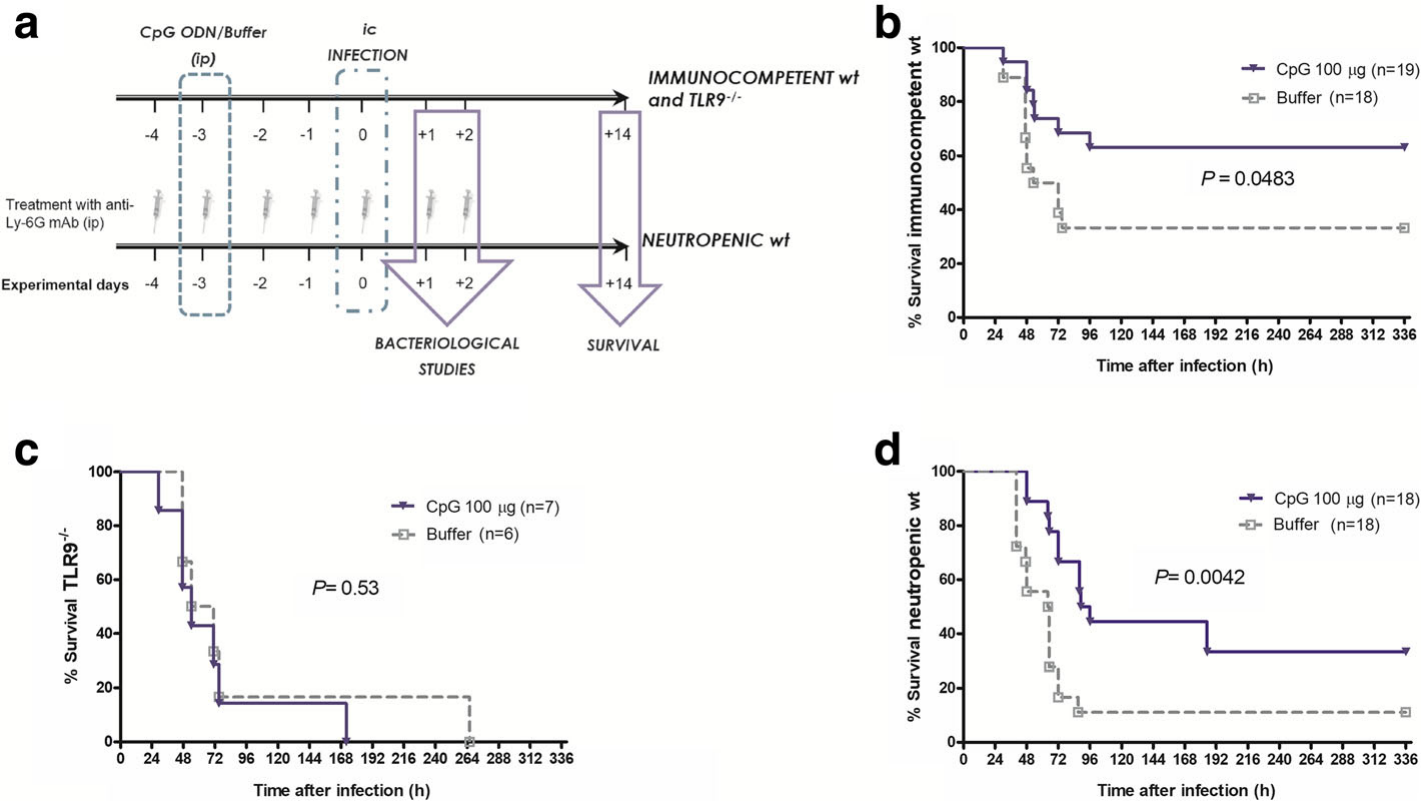
CpG ODN prolonged survival in CpG ODN-treated immunocompetent wild-type and neutropenic wild-type mice. (**a**) Treatment protocol. A single intraperitoneal (ip) CpG ODN was administered on day − 3, i.e., 3 days prior to infection. Mice were infected intracerebrally (ic) on day 0. Mice rendered neutropenic received anti-Ly-6G monoclonal antibodies ip from day − 4 until day 2. Kaplan-Meier curves in immunocompetent (**b**) and neutropenic (**d**) wild-type mice showed a prolonged survival (log-rank test, *P* < 0.05) and a tendency towards decreased mortality in CpG ODN-treated mice. (**c**) CpG ODN had no effect in Toll-like receptor 9-deficient mice (TLR9^−/−^)

A scheme of the course of the experiments is presented in Fig. 1a. The depletion of CD11b^+^Ly-6G^+^Ly-6C^int^ neutrophils was achieved by ip injection of 50 μg of anti-Ly6G monoclonal antibody (mAb, clone 1A8, BioXcell, West Lebanon, NH) [22]. A single injection of 50 μg of anti-Ly-6G MAb (1A8) caused a complete depletion of neutrophilic granulocytes in the blood at 24 h and 48 h after injection [22]. Each neutrophil-depleted mouse received a total of 7 daily injections [starting 4 days before infection (day − 4) until day + 2). All neutrophil-depleted mice received the same amount of anti-Ly-6G antibodies and were scored and weighed daily to evaluate any potential impact of the antibody injections. No weight loss and no behavioral abnormalities were noted as a consequence of the anti-Ly-6G antibody injections. To minimize stress to the mice, only the granulocyte-depleted animals received intraperitoneal antibody injections. In the present study, we did not compare granulocyte-depleted (i.e., mice which received an intraperitoneal antibody injection) with immunocompetent animals (i.e., mice which did not receive an intraperitoneal antibody injection). In a previous report [22], the effect of anti-Ly-6G antibody treatment to induce neutropenia was evaluated, and mice treated with IgG isotype antibodies were included in the experimental setting as controls. Therefore, in the present study, there was no need to treat immunocompetent mice with intraperitoneal pre-immune serum or isotype IgG.

The infection was performed at day 0. Meningitis was induced by inoculation of *S. pneumoniae* D39 intracerebrally (ic) into the superficial right frontal neocortex of anesthetized animals [23]. Immunocompetent wild-type and TLR9^−/−^ mice received 3 × 10^2^ colony-forming units (CFU)/mouse while neutropenic wild-type animals were challenged with 5 × 10^1^ CFU/mouse.

### Assessment of the clinical status of the animals

Mice were weighed and scored daily throughout the experiment. The clinical score was assessed as follows: 0, no apparent behavioral abnormality; 1, moderate lethargy; 2, severe lethargy; 3, unable to walk; 4, dead [23]. Mice were immediately sacrificed, when they were unable to walk (score 3) or when they had lost ≥ 20% of their body weight at study entry. Overall and free-of-symptom survivals were monitored until 14 days after infection. In the two different bacteriological studies, animals were sacrificed 24 h or 36 h after bacterial challenge.

### Sample processing

At the end points of bacteriological and survival studies, bacterial loads were determined in blood, cerebellum, and spleen homogenates. Cerebellum was analyzed instead of neocortex because in this experimental model the majority of bacteria are located in the CSF or attached to the meninges, and the ratio meninges-brain tissue is greater in the cerebellum than in the neocortex. In this model, bacterial concentrations in CSF and cerebellar homogenates are closely correlated [23]. Half of the cerebellum and half of the spleen were diluted 1:10 (w/v) in 0.9% NaCl, i.e., for each milligram of tissue, we added 9 μl of sterile 0.9% NaCl, and homogenized manually using a pellet pestle (Kisker Biotech, Steinfurt, Germany). Blood and tissue homogenates were serially diluted in 0.9% saline and plated on blood agar. If not stated otherwise, the detection limit was 100 CFU/ml. In bacteriological studies, approx. 100 μl of blood was taken 5 h before infection by puncture of the retro-orbital plexus. At the end of the experiment (24 or 36 h in bacteriological studies, or at the time of sacrifice during survival studies), after anesthesia with ketamine/xylazine, as much blood as possible (approx. 100–300 μl) was obtained by cardiac puncture. Blood not used for plating was stored for 30 min at 4 °C and then centrifuged at 3000×*g* for 10 min at 4 °C. Thereafter, serum and the rest of spleen and cerebellum homogenates were frozen at − 80 °C and later used for cyto- and chemokine analyses.

### Cyto-/chemokine measurements

Concentrations of IL-12/IL-23p40, macrophage inflammatory protein 1α (MIP-1α), and interferon-γ (IFN-γ) in serum and tissue homogenates were determined by DuoSet ELISA Development Kits (R&D Systems, Wiesbaden, Germany) according to the manufacturer’s instructions. In brief, the capture antibodies were diluted in PBS and allowed to bind to the plates overnight. Then, unbound capture antibody was washed away (buffer 0.05% Tween® 20 in PBS), and plates were blocked (buffer 1% BSA in PBS) and washed again (buffer 0.05% Tween® 20 in PBS). Thereafter, samples were added, and the analytes bound to the immobilized primary antibody. After further washing steps, the detection antibody was applied followed by the addition of streptavidin-horseradish peroxidase (1 ml diluted in 40 ml PBS containing 0.05% Tween® 20/0.1% BSA). After another washing step, a 1:1 mixture of Color Reagent A (H_2_O_2_) and Color Reagent B (tetramethylbenzidine) was added, and a blue color developed in proportion to the amount of analyte present. Color development was stopped by 2 N H_2_SO_4_ turning the color to yellow, and the absorbance of the color was measured at 450 nm. The sensitivity was 7.5 pg/ml for IL-12/IL-23p40 and MIP-1α, and 15 pg/ml for IFNγ.

### Immunohistochemical analyses

Immunohistochemistry for ionized calcium-binding adaptor molecule 1 (Iba-1), which stains all microglial cells, was used to identify microglial cells in paraffin-embedded, 2-μm coronal brain sections of mice sacrificed 24 h after infection. Neocortex and hippocampal formation were fixated in 4% paraformaldehyde in PBS, embedded in paraffin, and cut in 2-μm sections. Coronal sections located approx. 3 mm caudal of the bregma were incubated with polyclonal rabbit anti-Iba1 antibodies (dilution 1:400, Wako Chemicals, Neuss, Germany) overnight at 4 °C followed by incubation with biotinylated anti-rabbit IgG (dilution 1:200, GE Healthcare Europe, Freiburg, Germany), avidin-biotin peroxidase complex (VectastainTM ABC Kit, Vector Laboratories, Burlingame, USA) for 60 min each, and diaminobenzidine as chromogenic substrate (DAB Substrate, Roche, Mannheim, Germany). Sections were counterstained with hemalum. Stained sections were analyzed by a blinded investigator, and the number of Iba-1^+^ cells was determined in 8 fields of three different neocortical regions (containing the left and right cortical amygdaloid nucleus, auditory cortex, and visual cortex), and of the left and right hippocampal dentate gyrus (× 20 objective). For each animal, the numbers of microglial cells in the individual fields quantified were added and then divided by the number of scored fields [22].

In these 8 fields, in addition to the numbers of microglia, microglial morphology was quantified by a microglial activation score originally described by Kreutzberg [11] and adapted by different groups [24–26]: a score of 1 was assigned to cells with relative big somata but fine ramifications, a score of 2 to hypertrophic cells with thicker branches, a score of 3 to bushy cells, and a score of 4 to ameboid microglia without processes. The activation scores of each field assessed were added and then divided by the number of fields evaluated.

### Statistical analyses

Overall and symptom-free survivals were compared using the log-rank test. Differences between the two experimental groups were evaluated by Student’s *t* test in the presence or by Mann-Whitney *U* test in the absence of normal distribution. We directly compared CpG-treated versus buffer-treated wild-type immunocompetent and neutrophil-depleted mice, and CpG-treated versus buffer-treated TLR9-deficient mice. This experimental design, relying on comparisons of two groups only, produced meaningful results with a minimum number of animals. Comparing more than two groups with each other would have required *p* value adjustment for repeated testing and thereby would have strongly increased the number of animals necessary. Data sets with measurements below the limit of detection were always analyzed with non-parametric statistical methods. Here, measurements below the detection limit were assigned to the lowest rank(s). Correlation between bacterial titers and cytokine levels was analysed using the Spearman’s rank correlation coefficient (r_s_). For all analyses, the GraphPad Prism version 5 (GraphPad Software, San Diego, CA) was used, and a *P* value < 0.05 was considered statistically significant.

## Results

### CpG ODN induces TLR9-dependent prolongation of survival of immunocompetent animals with pneumococcal meningitis

CpG ODN given 3 days before infection prolonged the survival of immunocompetent wild-type mice (*P* = 0.048, log-rank test; Fig. 1b). Fourteen days after pneumococcal infection, 63% (12/19) of the animals receiving CpG ODN survived versus 33% (6/18) of the buffer-treated group (*P* = 0.10, Fisher’s exact test). Buffer-injected animals exhibited the first clinical symptoms significantly earlier than mice pre-treated with CpG ODN (*P* = 0.039, log-rank test). Pre-treatment with 100 μg CpG ODN did not protect TLR9^−/−^ mice against pneumococcal meningitis (*P* = 0.53, log-rank test; Fig. 1c). TLR9^−/−^ mice were more susceptible to the ic injection of 300 CFU/mouse than wild-type mice: the mortality rate was 100% both in CpG ODN- and buffer-treated TLR9^−/−^ animals.

### Pre-conditioning with CpG ODN delays the development of the first symptoms and prolongs the survival of neutropenic mice with pneumococcal meningitis

Mice rendered neutropenic after daily injection of anti-Ly-6G mAb were more susceptible to pneumococcal meningitis than wild-type mice. While 33% of the buffer-treated immunocompetent mice survived after ic challenge with 300 CFU *S. pneumoniae* D39/mouse, only 11% (2/18) of the buffer-treated neutropenic mice overcame the infection with 50 CFU *S. pneumoniae* D39/mouse. CpG ODN pre-conditioning was also effective in the immunocompromised host. Neutropenic animals primed with CpG ODN exhibited an extended survival compared to mice receiving buffer (*P* = 0.004, log-rank test; Fig. 1d). Accordingly, buffer-injected neutropenic animals developed the first clinical symptoms faster than CpG ODN-treated neutropenic animals (41 h versus 56.5 h, *P* = 0.05, log-rank test). As in immunocompetent mice, the mortality tended to be lower in CpG ODN-treated neutropenic wild-type mice (*P* = 0.23, Fisher’s exact test; Fig. 1d).

### Pre-treatment with CpG ODN reduces pneumococcal concentrations in the bloodstream and spleen of immunocompetent and neutropenic wild-type mice and in the brain of neutropenic wild-type mice

**Fig. 2:**
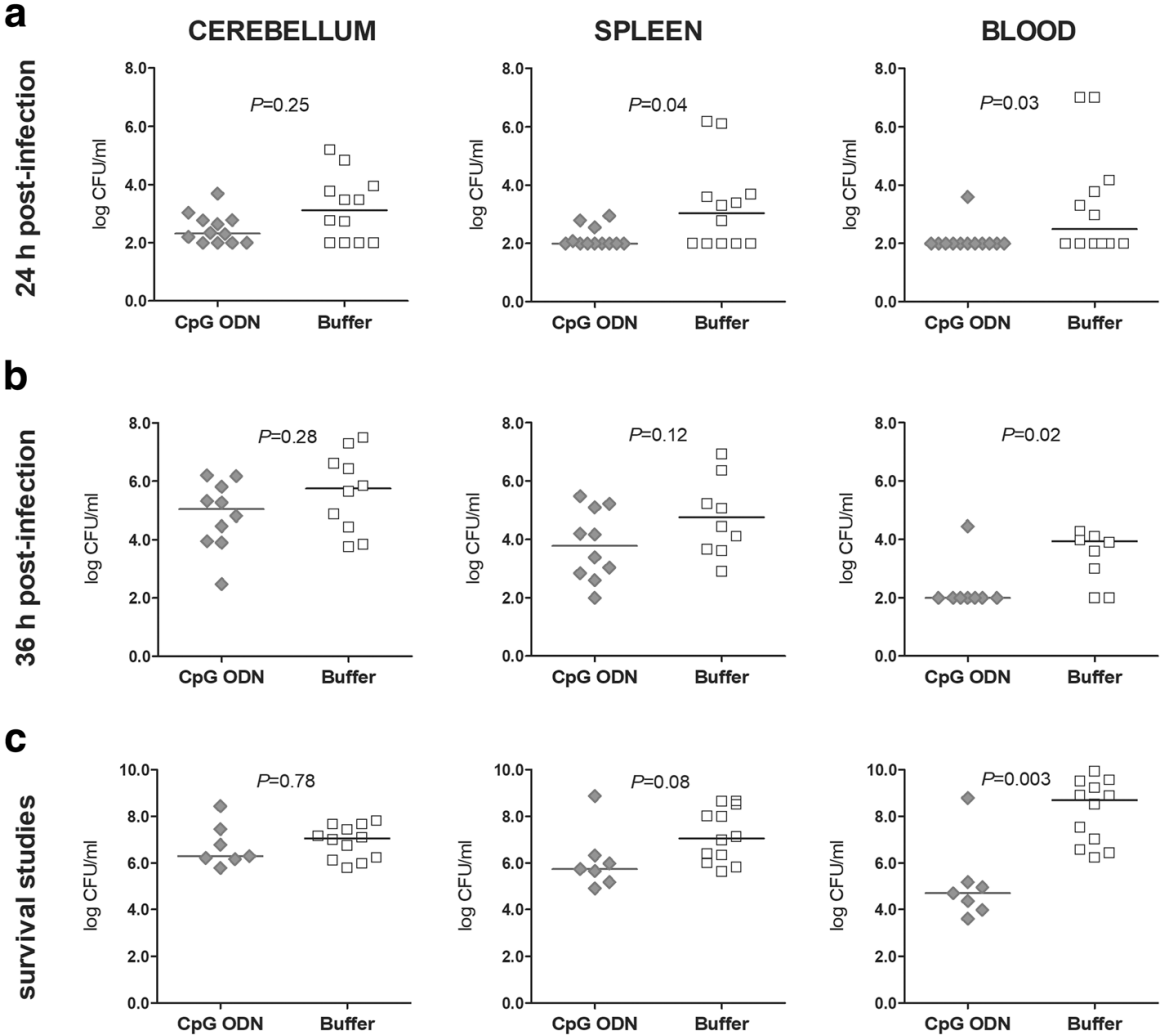
Pre-treatment with CpG ODN reduced the concentrations of pneumococci in the bloodstream in immunocompetent wild-type animals. Bacterial concentrations were determined in cerebellum and spleen homogenates as well as in the blood of immunocompetent mice from bacteriological studies with end point at (**a**) 24 h and (**b**) 36 h after infection. (**c**) Bacterial loads were also measured in immunocompetent mice which succumbed to the infection during survival studies. The detection limit was 10^2^ colony-forming units (CFU)/ml. Data are shown as medians, and each symbol represents an individual mouse. The Mann-Whitney *U* test was used to compare the bacterial concentrations in the CpG ODN- and the buffer-treated groups

**Fig. 3:**
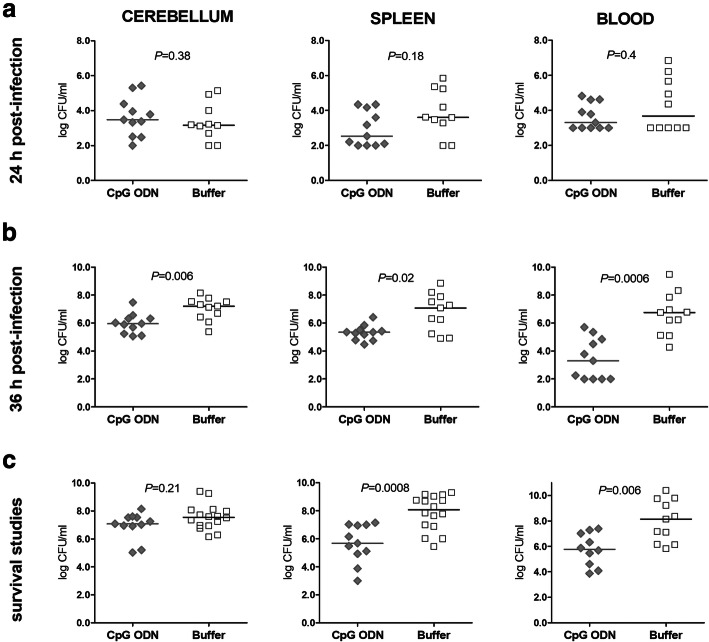
CpG ODN pre-treatment reduced pneumococcal concentrations in the brain, blood, and spleen in neutropenic wild-type animals. Bacterial loads were quantified in cerebellum and spleen homogenates as well as in the blood of neutropenic mice from bacteriological studies with end point at (**a**) 24 h and (**b**) 36 h after infection. (**c**) Bacterial concentrations were also assessed in neutropenic mice which succumbed to the infection during survival studies. Data are shown as medians, and each symbol represents an individual mouse. The detection limit was 10^2^ colony-forming units (CFU)/ml in all homogenate samples, and 10^2^ or 10^3^ CFU/mL in the blood. The Mann-Whitney *U* test was used to compare the bacterial concentrations in the CpG ODN- and buffer-treated groups

We next examined whether the effect exerted by CpG ODN was associated with an enhanced control of bacterial replication in the CNS and subsequently in the bloodstream and spleen. Therefore, bacterial concentrations in cerebellar and spleen homogenates as well as in blood samples were determined in immunocompetent (Fig. 2) and neutropenic (Fig. 3) wild-type animals. Bacterial loads were evaluated at the early phase of infection (24 h and 36 h after bacterial challenge) and during survival studies in those immunocompetent and neutropenic wild-type mice which succumbed to the infection (last immunocompetent and neutropenic wild-type animal sacrificed 96 h and 185 h post-infection, respectively).

There was a trend towards lower bacterial concentrations in the cerebellar homogenates of immunocompetent CpG ODN-treated wild-type mice at 24 h and 36 h, and in mice which had to be sacrificed thereafter, but no statistically significant differences between CpG ODN-treated and control animals. In granulocyte-depleted wild-type mice at 36 h after infection, bacteria in the cerebellar homogenates were lower after CpG ODN treatment (*P* = 0.006, Mann-Whitney *U* test; Fig. 3b). CpG ODN pre-conditioning significantly reduced bacterial growth in the bloodstream and in the spleen of both immunocompetent and immunosuppressed wild-type animals. Immunocompetent CpG ODN-treated wild-type mice showed lower bacterial loads in the blood throughout the infection (*P* ≤ 0.03, Mann-Whitney *U* test; Fig. 2a-c) compared to the control group. In the neutropenic host, CpG ODN immunization also reduced the amount of bacteria in the blood and spleen during pneumococcal meningitis at 36 h and in mice which succumbed to the infection (*P* ≤ 0.02, Mann-Whitney *U* test; Fig. 3b and c).

### CpG ODN induces a TLR9-dependent increase of microglial density at the early phase of infection

**Fig. 4:**
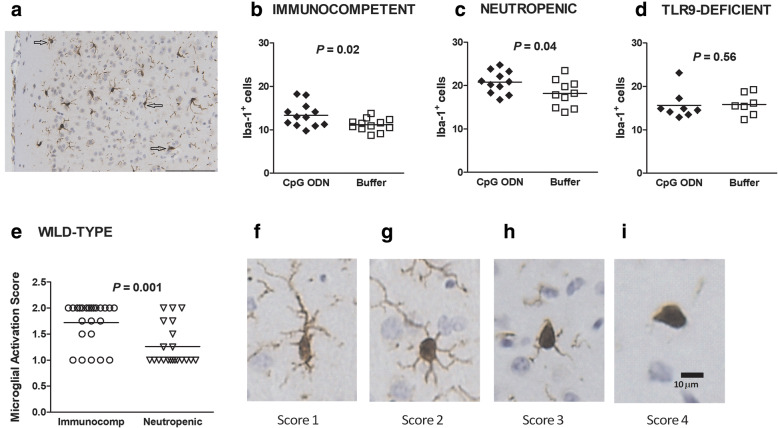
Microglial quantification and activation. (**a**) Microglial cells visualized by Iba-1 immunostaining were quantified, and their activation status was assessed in animals sacrificed 24 h after infection. Scale bar, 100 μm. Numbers of Iba-1-stained microglia in (**b**) immunocompetent and (**c**) neutropenic wild-type mice, and in (**d**) TLR9-deficient animals. (**e**) Microglial activation was stronger in infected wild-type immunocompetent than in infected granulocyte-depleted wild-type mice (*P* = 0.001). (**f-i**) Typical examples of the activation status of individual microglial cells: score 1—cells with relatively big somata but fine ramifications (**f**); score 2—hypertrophic cells with thicker branches (**g**); score 3—bushy cells (**h**); score 4—amoeboid microglia without processes (**i**)

A representative image of microglial cells stained with the anti-Iba1 antibody in the cortex of an infected buffer-treated mouse is depicted in Fig. 4a. The number of Iba-1^+^ cells was higher both in CpG ODN-treated immunocompetent and CpG ODN-treated neutropenic wild-type animals 24 h after infection compared to their respective control groups (*P* ≤ 0.04, Student’s *t* test; Fig. 4b,c). Microglial numbers in TLR9^−/−^ infected mice were not influenced by CpG ODN pre-treatment (*P* = 0.56; Student’s *t* test; Fig. 4d).

Overall, microglial activation (Fig. 4e-i) was stronger in immunocompetent than in granulocyte-depleted wild-type mice [median microglial activation score (25th/75th percentile) 2.0 (1.5/2.0) versus 1.0 (1.0/1.6), *P* = 0.001] 24 h after infection (Fig. 4e). No significant difference was noted in the microglial activation score of infected CpG-pretreated compared to infected control mice [immunocompetent wild-type mice 1.75 (1.0/2.0) versus 2 (1.8/2.0), *P* = 0.1; granulocyte-depleted wild-type mice 1.0 (1.0/1.5) versus 1.1 (1.0/1.8), *P* = 0.37)].

### CpG ODN induces Th1 responses in the spleen in both immunocompetent and neutropenic wild-type mice during pneumococcal meningitis

**Fig. 5:**
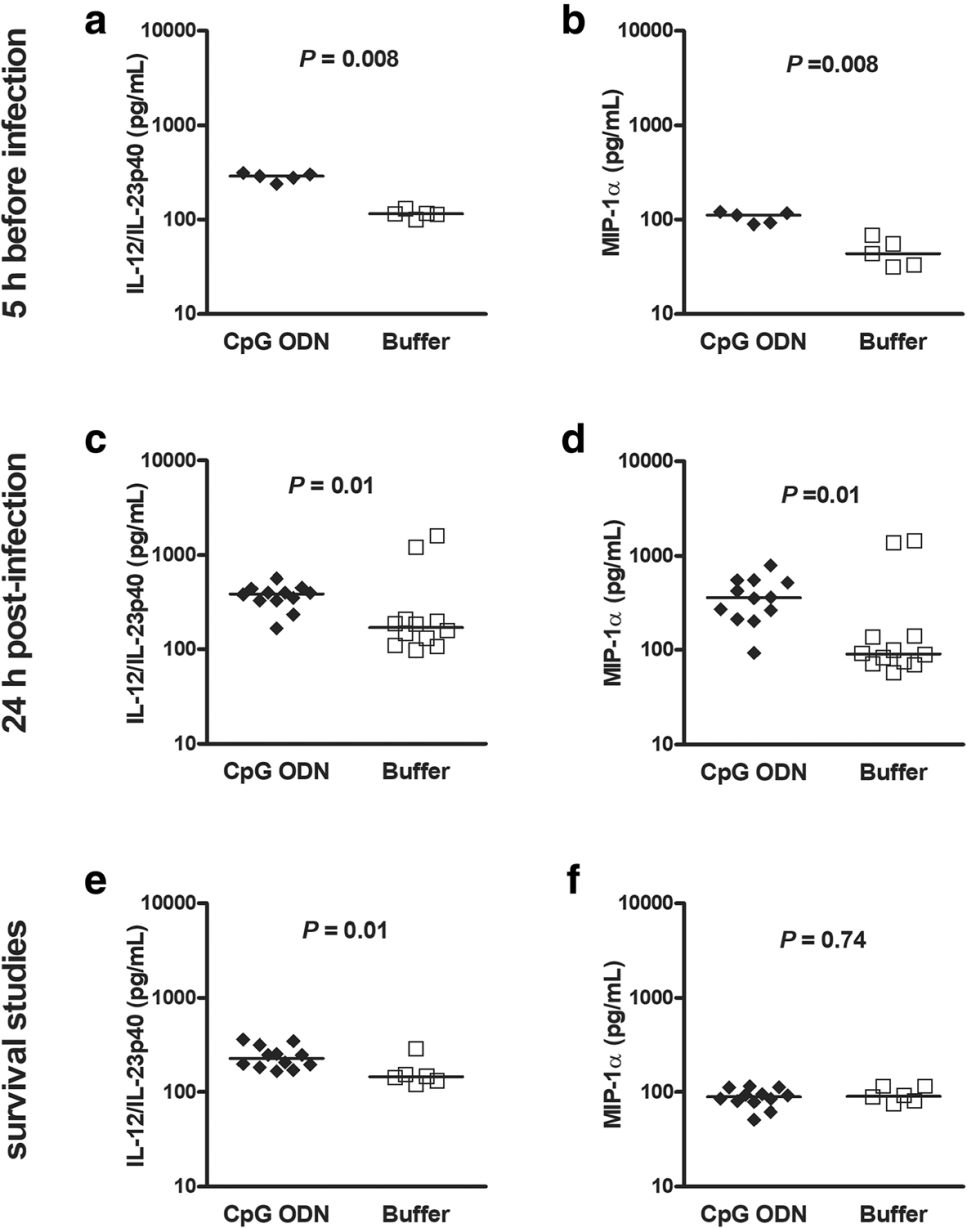
CpG ODN effects on IL-12/IL-23p40 and MIP-1α concentrations in spleen homogenates of infected immunocompetent wild-type mice. (**a**-**d**) In a set of bacteriological studies, spleen tissue from CpG ODN- and buffer-treated wild-type animals was obtained 5 h before (i.e., at this time point animals were uninfected) and 24 h after intracerebral challenge with *S. pneumoniae* D39. (**e**, **f**) In another set of experiments, spleens were obtained from wild-type animals which succumbed to the infection during survival studies. Data are shown as medians, and each symbol represents one immunocompetent mouse. CpG ODN- and buffer-treated groups were compared by Mann-Whitney *U* test. All concentrations measured were above the levels of detection

**Fig. 6:**
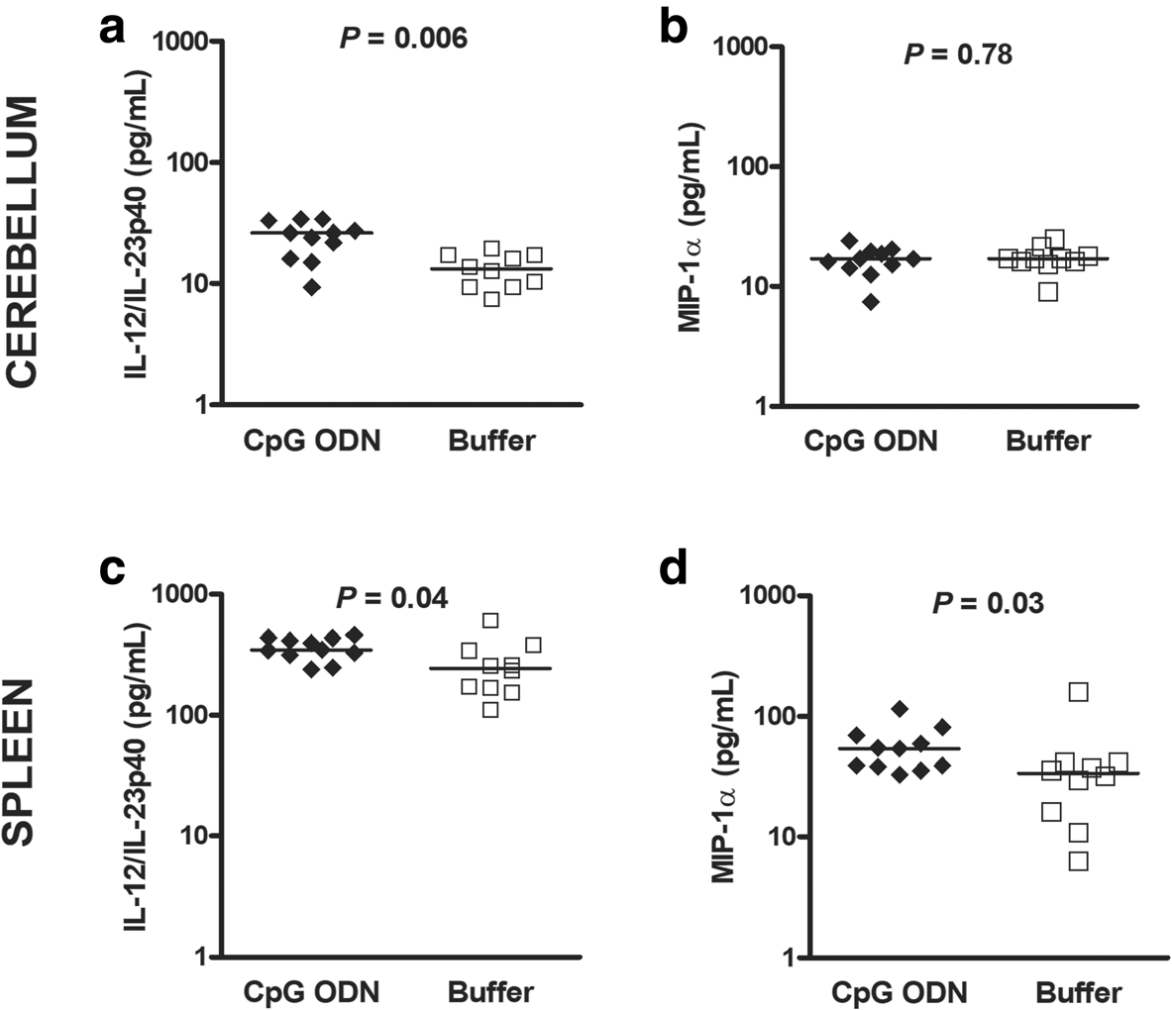
Concentrations of interleukin (IL)-12/IL-23p40 and macrophage inflammatory protein (MIP)-1α in cerebellum and spleen of CpG ODN-stimulated and control neutropenic wild-type mice 24 h after infection. (**a**, **b**) Cerebellum. (**c**, **d**) Spleen. IL-12/IL-23p40 concentrations were higher both in the cerebellum and spleen, whereas MIP-1α levels were only higher in the spleens of CpG ODN-treated mice (**a**): one sample from one mouse of the buffer group below the level of detection; (**b**): one sample from one mouse pre-treated with CpG below the level of detection; (**d**): one sample from one mouse of the buffer group below the level of detection

Adequate cytokine and chemokine responses are crucial to mount an efficient immune response against pathogens. Here, we measured IL-12/IL-23p40 and MIP-1α as typical drivers of Th1 responses [21] in the serum, spleen, and cerebellum of wild-type animals pre-treated with CpG ODN or buffer (Figs. 5 and 6). Three days after CpG ODN injection and 5 h before infection immunocompetent wild-type mice showed increased splenic levels of IL-12/IL-23p40 and MIP-1α compared to non-infected buffer-treated immunocompetent wild-type animals (*P* = 0.008, Mann-Whitney *U* test; Fig. 5a and b). Twenty-four hours after infection, concentrations of IL-12/IL-23p40 and MIP-1α in the spleen were higher in immunocompetent mice pre-conditioned with CpG ODN than in controls (*P* = 0.01, Fig. 5c and d). Remarkably, IL-12/IL-23p40 spleen concentrations in CpG ODN-treated wild-type animals remained elevated even at later post-infection times (*P* = 0.01, Fig. 5e). No differences in IL-12/IL-23p40 or MIP-1α concentrations were found in the cerebellum. IFN-γ concentrations were measured in the spleens of immunocompetent infected CpG ODN-treated and control wild-type mice 24 h after infection (*n* = 12/group). In both groups, most samples were below the quantification limit of 15 pg/ml (CpG ODN-treated: *n* = 7; controls: *n* = 10), i.e., the medians were both < 15 pg/ml (25th/75th percentile < 15/34.4 pg/ml and < 15/< 15 pg/ml; *P* = 0.37, Mann-Whitney *U* test).

In neutropenic-infected wild-type animals, IL-12/IL-23p40 levels were significantly elevated in both cerebellum and spleen homogenates of CpG ODN-pre-treated animals compared with buffer-injected mice 24 h after infection (Fig. 6a and c, *P* ≤ 0.04). Similarly, CpG ODN-preconditioned animals exhibited an increased production of MIP-1α in spleen compared to controls (Fig. 6d, *P* = 0.03). TLR9-deficient mice showed comparable amounts of IL-12/IL-23p40 and MIP-1α in the spleen 24 h after infection irrespective of CpG ODN treatment: IL-12/IL-23p40 levels in median (25th/75th percentiles) were 160.3 (121.1/182.8) pg/ml in CpG ODN-treated, and 226.8 (151.6/899.3) pg/ml in buffer-treated animals (*P* = 0.13). MIP-1α concentrations were 126.9 (75.1/155.9) pg/ml in CpG ODN-treated, and 116.1 (105.9/1652) pg/ml in buffer-treated mice (*P* = 0.46). Spleen IFN-γ concentrations were also comparable in CpG- (*n* = 11) and buffer-treated wild-type neutropenic animals (*n* = 10): medians (25th/75th percentiles) were 86.2 (46.2/107.2) and 79.6 (62.3/95.3) pg/ml, respectively (*P* = 0.86).

### CpG ODN-induced release of IL12/IL-23p40 inversely correlated with bacterial concentrations in the blood of immunocompetent and neutropenic wild-type mice

**Fig. 7:**
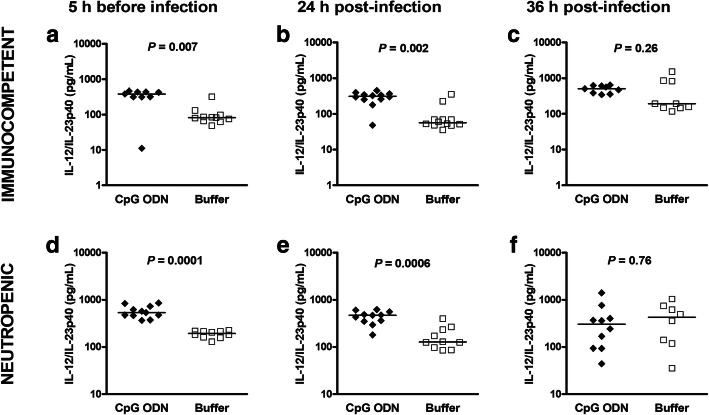
Serum levels of IL-12/IL-23p40 in uninfected and infected immunocompetent wild-type animals. (**a**, **b**, **d** and **e**) In bacteriological studies, serum samples from the same CpG ODN- and buffer-treated wild-type animals were obtained 5 h before and 24 h after intracerebral challenge with *S. pneumoniae* D39. (**c** and **f**) In another set of experiments, serum samples were obtained in wild-type animals 36 h after pneumococcal injection. Data are shown as medians, and each symbol represents an individual mouse. Data between CpG ODN- and buffer-treated groups were compared by Mann-Whitney *U* test. All concentrations measured were above the levels of detection. In **c**, **e**, and **f**, IL12/IL-23p40 inversely correlated with bacterial concentrations in the blood suggesting that IL12/IL-23p40 release was mainly caused by CpG ODN and less by the presence of bacteria

Levels of IL12/IL-23p40 were also quantified at different time points prior and after infection in the sera of immunocompetent and neutropenic wild-type animals. Three days after CpG ODN pre-treatment, 5 h before infection, non-infected immunocompetent and non-infected neutropenic wild-type animals exhibited higher concentrations of IL-12/IL-23p40 in serum compared to their respective buffer groups (*P* ≤ 0.007, Mann-Whitney *U* test; Fig. 7a and d). Serum concentrations of IL-12/IL-23p40 remained increased in CpG ODN-treated animals compared to buffer-injected mice 24 h after pneumococcal infection (*P* ≤ 0.002, Mann-Whitney *U* test; Fig. 7b and e). These cytokine levels among CpG ODN-primed animals were in the same range before and after infection. Circulating levels of IL-12/IL-23p40 were inversely correlated with bacterial densities in the bloodstream (neutropenic mice at 24 h: *r*_s_ = − 0.59, *P* = 0.06; at 36 h: *r*_s_ = − 0.79, *P* = 0.009; immunocompetent animals at 36 h: *r*_s_ = − 0.64, *P* = 0.07).

### CpG ODN effects on spleen size

**Fig. 8:**
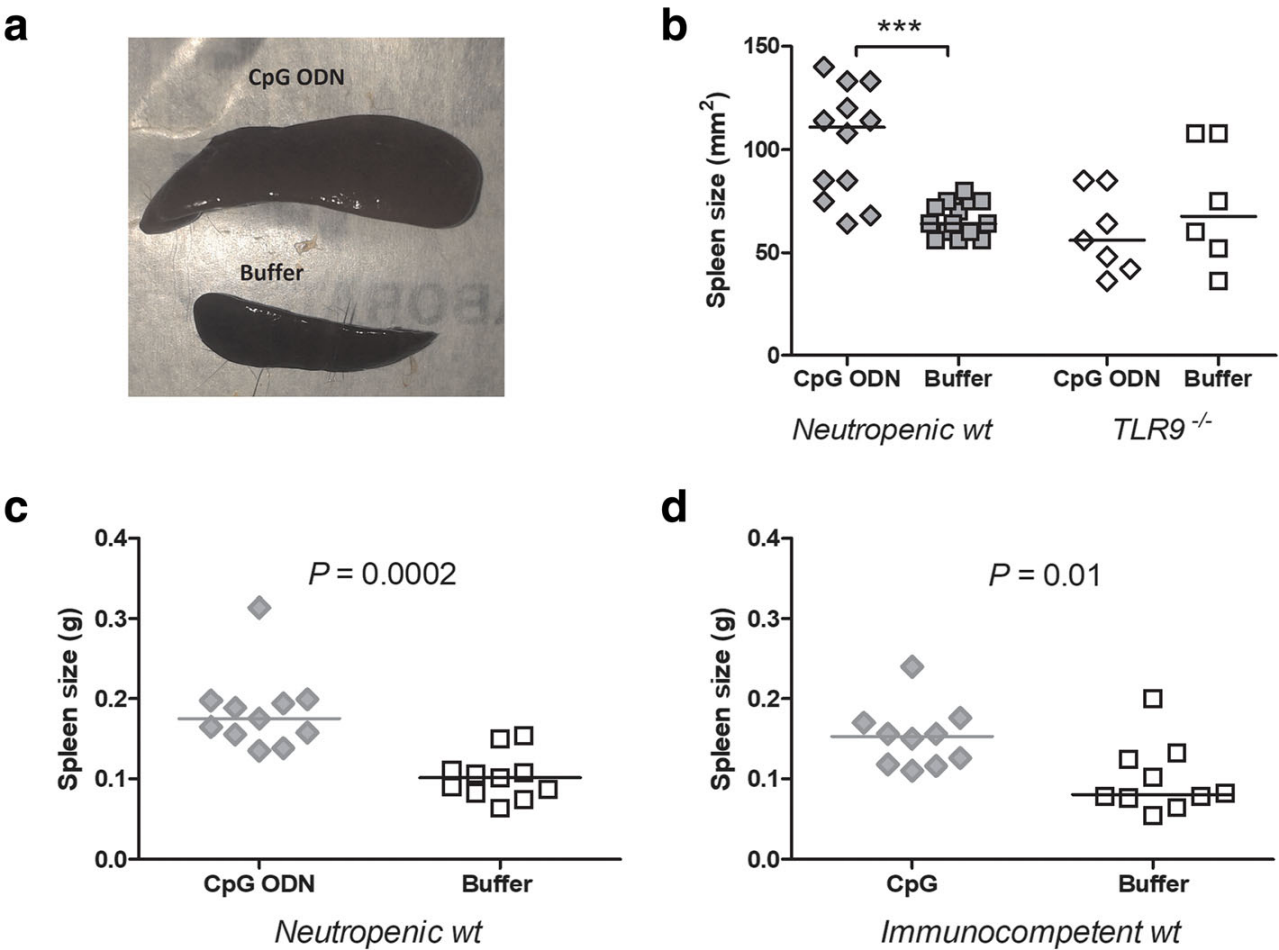
Spleen size in CpG ODN-stimulated and in buffer-treated control animals. (**a**) Photograph taken 36 h after infection, i.e., 4.5 days after CpG ODN stimulation, shows the strong enlargement of the spleen. (**b**) The spleen was enlarged in wild-type, but not in TLR9^−/−^ mice sacrificed after CpG ODN stimulation and infection. (**c**, **d**) Enlargement of the spleen was present in neutropenic and immunocompetent wild-type mice after CpG ODN stimulation and sacrificed after infection. The weight and age of the mice of the CpG ODN-stimulated and of the control group at the beginning of the experiment did not differ significantly indicating a similar size of the animals in the CpG ODN-stimulated and control group. When the spleen length was divided by the body weight of each mouse, the results of the statistical analysis remained unchanged being *P* values as follow (**b**), neutropenic wild-type mice: *P* < 0.0001; TLR-deficient mice: *P* = 0.45; (**c**): *P* = 0.001; (**d**): *P* = 0.007, Mann-Whitney *U* test). *** *P* ≤ 0.001

Transient splenomegaly after CpG ODN treatment was reported previously and was attributed to an increase and expansion of Gr1^+^CD11b^+^ myeloid cells in the spleen [27, 28]. We analyzed the spleens of CpG ODN and buffer-treated mice and observed that CpG ODN-related splenomegaly occurred in both immunocompetent and neutropenic wild-type animals but not in TLR9-deficient mice (Fig. 8).

## Discussion

Vaccination against all potential pathogens in groups of the population at a high risk of infection is an unrealistic goal. In particular for the pneumococcus, current vaccines target specific serotypes limiting their coverage. In the clinical setting, several reports suggest that vaccines and/or their adjuvants can confer a broader immune protection than the one they are designed for, according to a non-specific activation of the innate immune system. The most prominent example for the beneficial effect of heterologous immune activation is Bacillus Calmette-Guérin (BCG) vaccination: in children < 5 years in developing countries with a high prevalence of infectious diseases, BCG vaccination was associated with a reduction in all-cause mortality by 30–50% [29, 30].

We have previously shown that neutropenic mice receiving CpG ODN prior to infection were protected against *E. coli* K1 meningitis [21]. The aim of our present project was to show that CpG ODN do not only work with a Gram-negative, but also with a Gram-positive model organism. In adult HIV-patients, CpG ODN as an adjuvant successfully increased the immunogenicity of the 7-valent pneumococcal conjugate vaccine by enhancing antibody-independent cellular responses to pneumococcal polysaccharides [31, 32]. More recently, CpG ODN has been proven to enhance immunogenicity of DNA vaccines in a murine model of respiratory syncytial virus infection [33]. We hypothesize that CpG ODN has the potential to prime the innate immune response and to convey protection against different pathogens causing bacterial meningitis. In this and our previous study [21], CpG ODN priming reduced the bacterial concentration in the systemic circulation and prolonged the course of *S. pneumoniae* or *E. coli* meningitis. As a consequence of the relatively low number of mice studied in the two experimental settings (immunocompetent and neutropenic wild-type), however, the decrease of mortality achieved by CpG ODN treatment in the individual sets of experiments was not statistically significant. When all wild-type animals receiving CpG ODN in this study were compared to all wild-type animals receiving buffer, the mortality of CpG ODN-treated mice from *S. pneumoniae* was significantly lower than the mortality of control animals (CpG ODN-treated mice 18 alive/19 dead, controls 8 alive/28 dead; *P* = 0.0275, Fisher’s exact test). In the present and our previous study on *E. coli* [21], we opted for an ic challenge rather than an iv challenge, because an ic challenge transports a defined number of bacteria into the CNS producing a uniform course of the infection [23]. This allows to study in detail changes of bacterial concentrations and of the local immune response conveyed by microglial cells and invading white blood cells. The protective effect of CpG ODN observed in our intracerebral infection model will need to be expanded to other frequent bacterial meningeal pathogens, other strains of *S. pneumoniae* including those expressing pilus-1, and *E. coli*, and to meningitis models after intravenous or intranasal infection [10, 34–37].

In this model and in humans not receiving antibiotic treatment, host organisms survive when they are able to immediately clear bacteria which have entered the CNS. Once meningitis has been established, without antibiotic treatment the host will die. The reduction of mortality suggests that CpG ODN treatment will increase the probability of the host to clear an invasion of a small number of bacteria. Bacterial concentrations in this model do not only depend on bacterial growth, but also on the activity of phagocytes and on the exchange of bacteria between different compartments. There was a trend towards lower bacterial concentrations in the cerebellar homogenates of immunocompetent CpG ODN-treated wild-type mice at 24 h and 36 h, and in mice which had to be sacrificed thereafter, but no statistically significant differences between CpG ODN-treated and control animals in this setting. In granulocyte-depleted mice at 36 h after infection, bacterial concentrations in the cerebellar homogenates were lower after CpG ODN treatment (*P* = 0.006). These data (trend towards lower bacteria concentrations in the cerebellar homogenates of CpG ODN-treated immunocompetent wild-type mice at all time points and in granulocyte-depleted wild-type mice at 24 h and in survival experiments, significant difference in granulocyte-depleted mice at 36 h) suggest an inhibition of bacterial growth by CpG ODN not only in the systemic circulation, but also in the CNS. The decreased pathogen load in the bloodstream and in the spleen is clinically highly relevant because sepsis is a major cause of death in patients with pneumococcal infection [38].

The development of sepsis in the course of bacterial meningitis is not an unusual event: in the majority of cases, pneumococci reach the CNS via the bloodstream. In a minority of cases, bacteria directly enter the CNS through skull fractures/defects or (thrombosed) veins from infections in the vicinity of the brain (in particular sinusitis and mastoiditis). Here, bacteria do not reach the CSF across the blood-CSF barrier after septicemia, but they migrate directly from the infection site to the CSF and there reach high concentrations. In these cases, sepsis develops after meningitis: the bacteria causing the sepsis reach the blood from CSF (or—less likely—from the primary site of infection). Pneumococci (diameter approx. 1 μm) probably can pass the arachnoid granulations: in monkeys, *Saccharomyces cerevisiae* yeasts (3–6 μm), and goat (4 μm) and monkey (7 μm) erythrocytes passed the arachnoid granulations, whereas latex spheres of 6–12 μm were unable to pass them [39]. In man, free passage of particles up to 2 μm in diameter across the arachnoid granulations has been demonstrated [40]. We suggest that CSF containing high concentrations of bacteria is drained into the blood via the arachnoid granulations and thereby can cause sepsis. The decreased pathogen load in the circulation and the delay of the course of the infection by CpG ODN pre-conditioning is either caused by an inhibition of the spread of the infection to the bloodstream or by inhibition of the growth of bacteria in the blood, both mechanisms delaying the development of sepsis. This will broaden the therapeutic window, in which initiation of antibiotic treatment will rescue the host [41].

In clinical trials in humans, CpG ODNs “were reasonably safe when administered as vaccine adjuvants” [42]. Several studies, however, noted an elevation in the frequency and/or severity of local adverse events (injection site reactions) and systemic symptoms (including influenza-like symptoms, fever, and malaise) by CpG ODN-adjuvanted vaccines. Most of these adverse events were mild to moderate, started within 24 h of dosing and persisted for a few days only. No clear evidence has been found which may support concerns that CpG ODN may induce or worsen systemic autoimmunity [42].

The pharmacokinetic profile of oligonucleotides is similar after subcutaneous (sc), intradermal, ip, or intravenous administration, with highest maximum plasma concentrations after intravenous bolus infusion [43]. Since high maximum concentrations were not desired, we chose the ip route for the following reasons: (a) absorption of large molecules appears to be more reliable after ip than after sc injection [44], (b) larger volumes can be injected ip than sc, (c) mice immunized with heroin vaccine sc exhibited inferior anti-heroin titers compared to the ip and sc/ip coadministration injection routes [45], and (d) the ip route was effective in our previous study with *E. coli* [21].

TLRs are key molecules in the immune response against bacteria including *S. pneumoniae* [46–48]. Neutropenic wild-type mice were more susceptible to an intracerebral *E. coli* challenge than immunocompetent wild-type mice [22]. In neutropenic wild-type and TLR9-deficient mice, an inoculum of 1 × 10^4^ CFU *E. coli*/mouse was necessary to produce approx. 50% mortality, whereas in immunocompetent wild-type animals 1 × 10^5^ CFU/mouse achieved approx. 50% mortality [21]. Similarly, TLR9 deficiency increased mortality upon intranasal pneumococcal challenge in a murine pneumonia model [47]. Here, we demonstrated that TLR9 signalling is essential to convey the effect of CpG ODN in the host defence upon an intracerebral (ic) injection of *S. pneumoniae* D39. CpG ODN pre-conditioning protected both immunocompetent and neutropenic wild-type mice against *S. pneumoniae*, whereas TLR9-deficient mice were not protected.

In the brain, control of pneumococcal multiplication by microglia requires initial recognition of the different pathogen-associated molecular patterns of *S. pneumoniae*. During the early phase of meningitis, CpG ODN-treated immunocompetent and neutropenic wild-type animals displayed an enhanced proliferation/recruitment of microglial cells which was not observed in infected TLR9-deficient mice. In the initial phase of meningitis and encephalitis, the rapid and efficient elimination of pathogens by microglia is especially relevant, since neutrophils, which also contribute to pathogen elimination, initially are absent in the CSF and brain tissue.

Microglia can be activated by systemic inflammation by stimulation of the endothelial cells of the blood–brain barrier, by transport of pro-inflammatory chemo- and/or cytokines across the blood–brain barrier, by diffusion of pro-inflammatory bacterial products or cytokines across the fenestrated cerebromicrovascular endothelium of the circumventricular organs, or by autonomic afferent nerve fibers, especially the vagus nerve [12, 49]. Microglial activation was associated with an upregulation of TLRs, irrespective of the mode of stimulation [49]. In the present study, microglial activation as assessed by morphological criteria after CpG ODN administration was stronger in immunocompetent than in neutrophil-depleted wild-type mice suggesting that neutrophilic granulocytes contribute to the cross-talk with microglial cells across the blood–brain barrier.

Microglial proliferation in response to CpG ODN was observed both in immunocompetent and neutropenic wild-type mice, but not in TLR9^−/−^ mice. Injection of CpG ODN into peripheral lymph nodes primed lymphatic and systemic cytokine/chemokine release in uninfected and HIV-infected macaques [50]. Upon CpG ODN stimulation, uninfected immunocompetent and neutropenic wild-type animals rapidly developed elevated IL-12/IL-23p40 in the blood and spleen. Previous reports identified macrophages, lymphocytes, natural killer (NK) cells, and dendritic cells as sources of Th1-related cytokines in response to CpG ODN stimulation [51, 52]. Although we were unable to establish the exact mechanism of microglial activation by systemic administration of CpG ODN, we hypothesize that their activation possibly occurs by circulating IL-12/IL-23p40 and cross-talk of activated circulating white blood cells with microglia. Among the white blood cells, granulocytes appear not to play an essential role as mediators of protection because the effect of CpG ODN was also observed in granulocyte-depleted mice (present data and [21]).

It has previously been shown that both IL-12 and IL-23 are critical for antibacterial host defence [53]. In the present study, serum IL-12/IL-23p40 concentrations were continuously elevated throughout the infection in CpG ODN-treated immunocompetent and neutropenic wild-type animals for at least 17 days, and circulating levels of IL-12/IL-23p40 were inversely correlated with bacterial densities in the blood. Moreover, pre-conditioning with CpG ODN increased the concentration of MIP-1α in the spleen of immunocompetent and of neutropenic wild-type animals suggesting that neutrophils are not essential for MIP-1α release upon CpG ODN immunostimulation. IFN-γ, known to be a strong stimulator of the phagocytosis of bacteria by macrophages [54, 55], unlike in experimental *E. coli* meningitis [21] apparently did not play a major role in this model of *S. pneumoniae* meningitis, since spleen concentrations in most animals were low at 24 h after infection. CpG ODN also stimulates the release of other cytokines including IL-1, IL-6, IL-18, and TNF-α. Since the release of these cytokines is induced by the pneumococcal infection itself in humans and experimental animals, in the present set of experiments, we were unable to assess their contribution to the protective effect of CpG ODN [56–58]. Therefore, the gain of information concerning the protective effect of CpG ODN by measuring these cytokines is small. Finally, at the early phase of meningitis, CpG ODN-treated animals did not exhibit an increased recruitment of inflammatory monocytes and granulocytes into the CNS compared with buffer-treated animals. This observation indicated that in CpG ODN-primed animals the second immunostimulatory hit (i.e., the infection) did not exacerbate the inflammatory response in a detrimental manner neither at the site of infection nor in the systemic circulation.

## Conclusion

We proved the efficacy of CpG ODN pre-conditioning to counteract *S. pneumoniae* meningitis by rapidly strengthening both the systemic and the brain innate immune defense without boosting the general host inflammatory response. Because we demonstrated the efficacy of CpG ODN also in Gram-negative *E. coli* meningitis [21], our findings emphasize the potential of CpG ODN as a broad-spectrum adjuvant to prevent bacterial meningitis and delay its course thereby broadening the therapeutic window for starting an effective antibacterial therapy. Stimulation of TLR9 signalling “trains” the innate immunity [59] against Gram-positive and Gram-negative pathogens and provides cross-protection to common pathogens causing bacterial meningitis. By this mechanism, CpG ODN may convey protection against CNS infections in patients at high risk. In the present study, this effect was achieved by a single CpG ODN injection 3 days prior to infection. Further research is necessary to define how long the effect of a single CpG ODN injection lasts and whether protection will be prolonged and/or enhanced by repeated CpG ODN administrations.

## Data Availability

The datasets used and/or analyzed during the current study are available from the corresponding author on reasonable request.

## Abbreviations

CFU: Colony-forming units
CNS: Central nervous system
CpG ODN: Oligodeoxynucleotides containing unmethylated cytosine-guanine motifs
CSF: Cerebrospinal fluid
Iba-1: Ionized calcium-binding adaptor molecule 1
ic: Intracerebral(ly)
IFN-γ: Interferon gamma
IL: Interleukin
ip: Intraperitoneal(ly)
mAb: Monoclonal antibody
MIP-1α: Macrophage inflammatory protein-1α
NK cells: Natural killer cells
PBS: Phosphate-buffered saline
*r*_s_: Spearman’s rank correlation coefficient
TLR: Toll-like receptor
UMG: University Medical Center Göttingen
